# Response to reader comments on “clinical features of Hereditary Angioedema involving the gastrointestinal tract: A retrospective analysis”^☆^

**DOI:** 10.1016/j.waojou.2026.101405

**Published:** 2026-05-25

**Authors:** Haiyuan Ma, Danping Zheng, Baili Chen

**Affiliations:** Department of Gastroenterology, The First Affiliated Hospital, Sun Yat-sen University, 58 ZhongshanEr Road, Guangzhou, 510080, China

Dear Editor,

We appreciate the reader's interest in our article^1^ and thank them for the careful review and constructive comments. We are pleased to provide the following clarifications.

Regarding the clinical categorization, “mixed-type” refers to patients experiencing skin, gastrointestinal, and laryngeal edema with similar frequency and severity, whereas “predominant-type” identifies a single anatomical site as the primary attack pattern. In our study, we employed a dual-analytical approach: we first evaluated edema by anatomical site across the cohort, then further classified patients based on their self-reported predominant pattern (considering both frequency and severity). Crucially, experiencing edema at a specific site did not automatically define a patient as site-predominant; for instance, while 53 patients reported gastrointestinal involvement, only 32 met the criteria for gastrointestinal-predominant. This distinction between site-specific descriptive analysis and predominant-pattern subgrouping enhances both statistical clarity and clinical interpretability.

With respect to Supplementary Table 1, we apologize for the ambiguity. The designation “N = 53” refers to the subgroup of patients with gastrointestinal edema, rather than the number of patients with comorbidities. Among these 53 patients, 31 (58.5%) had at least 1 comorbidity. Because some patients had multiple comorbid conditions, the sum of individual comorbidities exceeds the number of affected patients.

Regarding the data on misdiagnosis and unnecessary surgery, among the 53 patients with gastrointestinal edema, 39 patients (73.6%) were misdiagnosed, and 12 patients (22.6%) underwent unnecessary surgery due to misdiagnosis. Figure 2d illustrates the distribution of surgical procedures among these 12 patients. Specifically, 10 patients (83.3%) underwent appendectomy, 3 patients (25.0%) underwent laparoscopic exploration, and 1 patient (8.3%) underwent laparotomy for presumed intestinal volvulus. Because some patients underwent more than 1 surgical procedure, these proportions are not mutually exclusive.

Regarding “hiccup” in Figure 2b, hiccup is involuntary contraction of the diaphragm mediated by a reflex arc involving the phrenic nerve, vagus nerve, and central nervous system. Physiologically, they are considered a respiratory reflex phenomenon. However, in clinical practice, hiccups are frequently triggered by gastrointestinal stimuli and are therefore often described as gastrointestinal-related symptoms. We agree that this dual nature may lead to ambiguity and appreciate the reader for raising this point.

Regarding C1-INH levels in Figure 3, the C1-INH level refers to the antigen level (concentration) rather than functional activity, as described in the Methods section.

Regarding “spontaneous” in Supplementary Table 2, we agree that “spontaneous” is not a trigger per se. In this study, the term was used to indicate episodes with no identifiable trigger, and we acknowledge that this wording may be misleading.

Regarding the potential influence of treatment, we would like to clarify that all clinical characteristics and laboratory parameters reported represent baseline data obtained prior to treatment initiation, and are therefore unlikely to be influenced by therapies such as lanadelumab, danazol, or icatibant.

Once again, we sincerely thank the reader for the careful and constructive comments. These observations have helped us to clarify several important aspects of our study and improve the transparency of our reporting. We greatly appreciate the opportunity to provide these clarifications.

## Author contributions

All authors made substantial contributions to the conception, design, drafting and revision of this manuscript and gave final approval of the version to be published.

## Ethics statement

The research was approved by the Research and Ethics Committee of the First Affiliated Hospital of Sun Yat-sen University ([2025] 170). Informed consent has been obtained from all patients prior to the study, and patients have signed informed consent forms for the release of their data.

## Author's consent

All authors agreed to publication of this work.

## Declaration of Generative AI and AI-assisted technologies in the writing process

Nothing to disclose.

## Funding

The authors acknowledge financial support from Guangdong Weiji Medical Development Foundation (K-20240204).

## Declaration of competing interests

There are no competing interests for any author.
